# Neural Mechanisms of Reward-by-Cueing Interactions: ERP Evidence

**DOI:** 10.3389/fnhum.2021.608427

**Published:** 2021-05-03

**Authors:** Xian Li, Meichen Zhang, Lulu Wu, Qin Zhang, Ping Wei

**Affiliations:** ^1^Beijing Key Laboratory of Learning and Cognition, School of Psychology, Capital Normal University, Beijing, China; ^2^Beijing Advanced Innovation Center for Imaging Technology, Capital Normal University, Beijing, China

**Keywords:** inhibition of return, target-reward association, event-related potential, reward, attention

## Abstract

Inhibition of return (IOR) refers to the phenomenon that a person is slower to respond to targets at a previously cued location. The present study aimed to explore whether target-reward association is subject to IOR, using event-related potentials (ERPs) to explore the underlying neural mechanism. Each participant performed a localization task and a color discrimination task in an exogenous cueing paradigm, with the targets presented in colors (green/red) previously associated with high- or low-reward probability. The results of both tasks revealed that the N1, Nd, and P3 components exhibited differential amplitudes between cued and uncued trials (i.e., IOR) under low reward, with the N1 and Nd amplitudes being enhanced for uncued trials compared to cued trials, and the P3 amplitude being enhanced for cued trials vs. uncued trials. Under high reward, however, no difference was found between the amplitudes on cued and uncued trials for any of the components. These findings demonstrate that targets that were previously associated with high reward can be resistant to IOR and the current results enrich the evidence for interactions between reward-association and attentional orientation in the cueing paradigm.

## Introduction

Recently, a wealth of studies have investigated the relationship between reward and spatial attention using the spatial cueing paradigm (Engelmann and Pessoa, 2007; Engelmann et al., 2009; Baines et al., 2011; Bucker and Theeuwes, 2014, 2016). These studies have demonstrated that the expectation of reward serves as a form of incentive motivation, which can be considered “pro-active” or a global preparatory strategy, leading to better performance (for reviews, see Chelazzi et al., 2013; Pessoa, 2015; Krebs and Woldorff, 2017). For instance, studies by Engelmann and Pessoa (Engelmann and Pessoa, 2007; Engelmann et al., 2009) showed when participants were informed by explicit instructions that they would receive a monetary reward contingent upon their performance, their detection of a target following a spatial cue was improved.

However, little is known about the effect of reward association on spatial attention (but see Bucker and Theeuwes, 2016). Reward association does not allow for preparation in expectation of an extra incentive. Rather, participants have to react “on the fly” to changing reward contingencies (e.g., Krebs et al., 2012; Bucker and Theeuwes, 2016). Such a learned association has been shown even to affect performance on later trials when the reward is no longer at stake (Della Libera and Chelazzi, 2006, 2009; Hickey et al., 2010; Anderson and Yantis, 2012, 2013; Hickey and Peelen, 2015). Without any pre-task cues, reward effects in these stimulus-reward association paradigms rely more on reactive or even automatic processes rather than on preparatory mechanisms (Krebs and Woldorff, 2017). The present study was designed to investigate the effect of reward association on the inhibitory process in spatial orienting, which is known as inhibition of return (IOR).

Posner and Cohen (1984) first reported the phenomenon that responses were slower for targets presented at a previously cued location with long cue-target stimulus onset asynchrony (SOA), which serves an important adaptive role for preventing repeated searching for information in a location to which attention has already been captured (i.e., the cued location) and biasing the attentional system toward novel locations (Posner et al., 1985; Klein and MacInnes, 1999; Klein, 2000; Lupiáñez et al., 2006). Based on this idea, Klein and MacInnes (1999) described IOR as a “foraging facilitator” that energizes the organism’s ability to scan the environment and detect potentially meaningful events. Over the years, a wealth of studies have suggested that IOR is a “blind” mechanism, i.e., an automatic process that is not affected by participants’ personal beliefs or goals (e.g., Theeuwes, 1994, 2010; Handy et al., 1999; Theeuwes and Godijn, 2002; Taylor and Therrien, 2005; Wang et al., 2010).

Several recent studies have been conducted to investigate the effect of reward on IOR. Some of these studies (Engelmann and Pessoa, 2007; Engelmann et al., 2009; Bucker and Theeuwes, 2014) informed participants about the reward/punishment contingencies at the beginning of each block, and others manipulated the cue-related reward associations (Bucker and Theeuwes, 2016). In one study by Bucker and Theeuwes (2014), reward information—either high (50% chance of winning € 1.00) or low (50% chance of winning € 0.10)—was provided before the start of each block in a typical exogenous cueing paradigm with short and long SOAs. Their results revealed that high rewards enhanced IOR. That is, with a long SOA, reaction times (RTs) were significantly slower in cued locations than in uncued locations under the high-reward condition, whereas no difference was found between cued and uncued trials under the low-reward condition. Bucker and Theeuwes’ explanation was that high-reward motivation elicited reorienting of attention away from the initially cued location to bias the search for new locations better. Interestingly, when the researchers used the same exogenous cueing task, but with the peripheral cues shown in colors associated with appetitive, aversive, and neural outcomes (Bucker and Theeuwes, 2016), RTs in the long-delay condition were shorter for cued than uncued trials when the cue was associated with high reward so that attention was then oriented to the cued location under the high-reward condition. Although these results provide preliminary evidence that IOR can be influenced by participants’ personal goals and behavioral outcomes, our knowledge about the mechanisms underlying the influence of reward on IOR is incomplete. To date, no study has been conducted to investigate the effect of target-related reward associations on IOR. With the same spatial cue, it is of theoretical importance to examine whether targets with different reward associations in the past can produce differential modulation of the inhibitory processes at the cued location.

The present study aimed to examine whether the process of prioritizing reactive attention, led by target-reward association, is subject to the automatic process of IOR, and to investigate the underlying neural mechanism of this reward-by-cueing interaction using event-related potentials (ERPs). In the current study, high- and low-reward associations with different colors were learned during a learning phase (e.g., Della Libera and Chelazzi, 2006; Hickey et al., 2010; Kristjánsson et al., 2010; Anderson et al.,2011a,b, 2013; Hickey and Peelen, 2015), whereas, during the test phase, the target (but not the spatial cue) was shown in the previously reward-associated color, though reward was no longer at stake. The separation between the learning and test phases was used to exclude the motivational factor encouraged by reward, so as to minimize top-down control and maximize the automatic attentional guiding process during the test phase. We hypothesized that when the target was previously associated with different levels of reward, attention would be drawn to the high-reward associated target faster than to the low-reward associated target. Therefore, we predicted if target-reward association is resistant to IOR, even though we might observe differences between cued and uncued trials (i.e., IOR) in the low-reward condition, such differences should be reduced or possibly even eliminated under the high-reward condition. However, if target-reward association is subject to IOR—as it is an automatic process—the difference between cued and uncued trials should be observed for both the low- and high-reward conditions.

The present study used two types of tasks (i.e., a localization task and a color discrimination task) in order to examine whether and how the expected interaction between reward and cueing would be manifested across different task requirements. In the former task, participants were asked to report the location (either left or right) of the target, while in the latter, they were asked to discriminate the color of the target. Earlier studies have shown that IOR can be affected by task type (e.g., Kwak and Egeth, 1992; Terry et al., 1994; LupiaìnÞez et al., 1997; Pratt et al., 1997; Cheal et al., 1998; Martín-Arévalo et al., 2013); hence, a comparison of the expected interactive effects of reward and IOR between tasks are of interest. Moreover, by using ERP recordings the present study allowed us to reveal the neural mechanism underlying the interaction between the target-reward association and spatial cueing (i.e., IOR). The present study examined IOR with sensory/perception and attentional components in the visual-spatial attention field [see Pan et al. (2017) for a recent review], with the electrophysiological components of interest being the P1, N1, Nd, and P3.

Based on the attentional reorienting hypothesis assumed by most researchers in the field, IOR is led by an inhibited attentional reorienting to the target location, which may produce modulations at different stages of processing. Under this framework, the early target perceptual processing indexed by the P1 and N1 components are predicted to show a reduction for cued vs. uncued trials (McDonald et al., 1999; Prime and Jolicoeur, 2009; Satel et al., 2013, 2014). Moreover, Satel et al. (2013) performed a correlation analysis on the mean IOR scores and the P1 and N1 modulation effects (i.e., P1 and N1 respective reductions for cued vs. uncued trials) across 19 experiments (Satel et al., 2013). The results showed that the P1 and N1 reductions for cued (vs. uncued) trials were associated with increased IOR scores (*r* = −0.60 for P1; *r* = −0.52 for N1; both *ps* < 0.05; two-tailed). This is to say that the slower it is to process a target at the previously cued location, the more likely it is the P1 component will be impaired on cued trials. However, many studies have found IOR effects without P1 modulation (e.g., Prime and Ward, 2006; Wang et al., 2012), while others have found P1 modulation without a behavioral IOR effect (e.g., Wascher and Tipper, 2004; Chica and Lupiáñez, 2009; Martín-Arévalo et al., 2014). Similarly, the N1 modulation effect is also inconsistent across the literature (Hopfinger and Mangun, 1998; Prime and Ward, 2004; Prime and Jolicoeur, 2009; Satel et al., 2014). While researchers have proposed several hypotheses for why P1 and N1 modulation was inconsistent with the behavioral IOR effect in those studies, some researchers suggest that they may not be stable electrophysiological markers of IOR (Prime and Ward, 2006; Jones and Forster, 2012, 2014; Satel et al., 2014).

The later Nd and P3 components were not well-characterized under the same framework, with inconsistent predictions and findings (see Martín-Arévalo et al. (2016) for review]. On the one hand, if they were to index the post-perceptual resource allocation, processing of the attended stimuli, and/or decision processing, we may expect to see a reduced Nd and P3 for cued trials compared to uncued trials. On the other hand, if the P3 component reflects target expectancy or psychological surprisal, we would expect to see an enhanced P3 component for cued trials. The Nd component was observed in most experiments, with a reduced amplitude for cued location (vs. uncued) used to reflect the IOR effect (e.g., Satel et al., 2013, 2014). Moreover, several studies have found a significant association of IOR with P3 enhancement for cued compared to uncued location trials (McDonald et al., 1999, Experiment 1; Prime and Jolicoeur, 2009), though no modulations have been reported in other studies (e.g., Hopfinger and Mangun, 2001; Chica and Lupiáñez, 2009; Martín-Arévalo et al., 2014). More often than not, studies have not examined or reported the P3 and/or Nd components because they were thought to be less relevant to IOR. However, in light of the above-mentioned hypothesis and evidence, the Nd and P3 components may actually reflect certain stages of processes that underlie IOR.

Overall, we hypothesized that the automatic mechanisms of attention prioritization guided by reward association would effectively modulate IOR. More specifically, we predicted that a high-reward associated target would no longer be subject to IOR. Therefore, an interaction between reward level and cueing effect should be observed. We may expect to observe amplitude differences between cued and uncued trials specifically under the low reward-condition on the P1, N1, Nd, and/or P3 components, whereas such an effect should be diminished under the high-reward condition. Moreover, while task types may or may not interact with the association between reward and cueing effects, according to prior studies, the different task demands may individually interact with the cueing effect or the reward effect.

## Materials and Methods

### Participants

Twenty-four healthy young adults took part in this study. All participants were right-handed with normal or corrected to normal vision, and had no known cognitive or neurological disorders. Participation was voluntary, with payment comprised of a base value and an additional value according to task performance upon completion of the study. Two participants were stopped from finishing the task because they were unable to comply with task demands (more than 20 consecutive trials skipped without any response); another participant was excluded due to poor accuracy (dropped below 75% in reporting the target location/color), leaving insufficient numbers of trials per condition for further analysis; and one other participant was excluded from the analysis due to excessive horizontal saccades during trials (>30% of the trials contained horizontal saccades). Data from 20 participants (mean age = 23, age range = 19–25, SD = 2.06; 12 females and eight males) were included in the further analyses. Our study was approved by the Ethics Committee of the School of Psychology at Capital Normal University, and all participants gave informed consent prior to the experiments, in accordance with the Declaration of Helsinki.

### Materials

Stimuli were presented on a 17-inch CRT display (black background), with a resolution of 1,024 × 768. The software Presentation^1^ was used to present stimuli and record responses. Participants were seated comfortably at a distance of 65 cm from the screen in a quiet room, with light adjusted to fit. Participants were asked to keep their head and chin still throughout the experiment.

During the learning phase, the target stimuli included four types: red round, red triangle, green round, and green triangle. A particular color was associated with high- or low-reward probability. Specifically, for half of the participants, red was associated with a higher possibility of reward: when the target was red, a correct response could result in an 80% chance of high reward (feedback: “¥50”) and a 20% chance of low reward (feedback: “¥5”); whereas, when the target was green, a correct response would lead to an 80% chance of low reward (feedback: “¥5”) and a 20% chance of high reward (feedback: “¥50”). The assignment of reward color was reversed for the other half of the participants.

During the test phase, three boxes (2° × 2°) were displayed in parallel. The center-to-center distance between the central box and each peripheral box was 5° of visual angle. Participants were required to fixate on the central box, while the sudden bolding of the outer frame in one of the two peripheral boxes served as a cue. The target could be either a red or green square presented in one of the peripheral boxes.

### Procedures

A task type (localization/color discrimination) × reward level (high/low) × cue validity (cued/uncued) within-participant factorial design was used.

In the rewarded learning phase, a fixation cross “+” (0.4° × 0.4°) appeared at the start of each trial for 600–800 ms, followed by the target stimulus (4.5° × 4.5°) presented at the center of the screen until the participant responded. Participants were asked to determine the shape of the target, i.e., click the left button of the computer mouse for round, and the right button for triangle. Then, a feedback screen with “¥50” or “¥5” was presented according to the assigned reward probability for 800 ms. After an inter-trial interval of 1,000–1,500 ms with a blank screen displayed, the next trial started.

Before the start of the learning phase, participants underwent 20 practice trials. The formal rewarded learning phase was divided into eight blocks, with 40 trials in each block, and thus, a total number of 320 trials, with 160 high-reward trials and 160 low-reward trials. The base pay for the experiment was 75 Chinese Yuan, with additional pay commensurate with participants’ performance and the reward level of the given trials. Specifically, participants were informed that the accumulated amount of experimental money presented in the feedback screen would be translated to real money at the end of the experiment. The total pay was between 90 and 100 Chinese Yuan for each participant.

**FIGURE 1:**
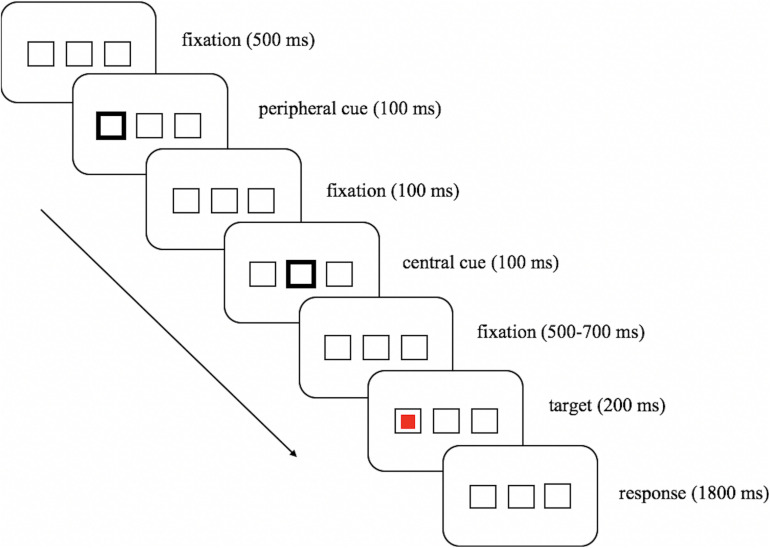
Example of the trial sequence in the test phase.

In the test phase (see Figure 1), an initial screen with three parallel boxes (2° × 2° for each box) appeared throughout each trial. The center-to-center distance between the central box and each peripheral box was 5° of visual angle. After the first 500 ms, a peripheral cue signified by the brightening of the outer frame of one of the two lateral boxes was presented for 100 ms, which was followed by a 100 ms peripheral-central cue interval, and then another 100 ms central cue signified by the brightening of the outer frame of the central box followed. After a variable interval of 500–700 ms, the target (red or green square) appeared at either the cued location or the uncued location for 200 ms, and participants were asked to respond within 2,000 ms, starting from the display of the target. Note that the target presented equally often at the cued and the uncued location, thus the peripheral cue did not predict the target location.

In the location task, participants needed to indicate the target location by clicking the left key of the mouse for a target on the left side, and the right key for a target on the right side of the screen. In the color discrimination task, half of the participants were asked to indicate the target color by left-clicking the mouse for red and right-clicking it for green, while the other half of the participants did the reverse. Before the start of the official test phase, participants underwent 20 practice trials for each task, during which subjects familiarized themselves with the task demands and practiced fixating on the central box. The assignment of the two task types was sequenced and balanced across participants. Each task type had 10 blocks, and each block had 32 trials. Thus, each experimental condition had 80 trials.

### EEG Recording and Analysis

The electroencephalogram (EEG) was recorded by a 62 Ag/AgCl electrodes cap, according to the extended 10/20 system, and continuously sampled at 500 Hz, with a bandpass filter of 0.05–100 Hz. Vertical and horizontal EOGs were recorded with two pairs of electrodes: one placed above and below the left eye, and another 10 mm from the lateral canthi. EEG signals were referenced to the left mastoid during recording and re-referenced offline to the average of the left and right mastoid recordings. The ground electrode was placed between FPz and Fz, and the impedance of the electrodes was kept under 5 kΩ throughout the EEG recording.

Data were collected with Neuroscan acquisition software, and EEG data processing was performed off-line using Neuroscan 4.5 software. All segments were checked offline for artifacts (blinks, saccades, and drifts). Trials with horizontal eye movements in the interval from cue onset to 400 ms post-target onset were rejected (<2.5% in each condition; Wascher et al., 2015). The remaining eye movements and blinks (that almost exclusively occurred in between trials) were corrected using the ocular artifact reduction algorithm in the Neuroscan v.4.5 software package. The remaining trials with artifacts that exceeded ±75 μV were excluded from the analysis. Artifact-free EEG was then segmented into epochs, starting from 100 ms (as the baseline correction) pre-target onset to 1,000 ms post-target onset and averaged separately for each participant, with each condition having over 60 valid trials.

Event-related potential responses recorded during the test phase were analyzed. Corresponding EEG activity for the correct responses under each condition was averaged to extract the ERP data for each of the 16 conditions: task type (localization, color discrimination) × reward level (high, low) × cue validity (cued, uncued) × target position (left, right). Based on the ERP literature on spatial attention, task preparation, and reward processing, the current study selected CP1/CP2 (e.g., Zimmer et al., 2015), P1/P2 (e.g., Schevernels et al., 2014), PO3/PO4 (e.g., Schevernels et al., 2014; Feldmann-Wüstefeld et al., 2015; Zimmer et al., 2015), and PO7/PO8 (e.g., Prime and Ward, 2004; Kiss et al., 2009; Feldmann-Wüstefeld et al., 2015). More specifically, in the study by Zimmer et al. (2015), the authors selected the cluster of CP1, CP2, P3, P4, PO3, and PO4 to study how motivation can guide spatial attention. In another study, Feldmann-Wüstefeld et al. (2015) used the electrode cluster of PO3, PO4, PO7, and PO8 to investigate the relationship between associative learning and visual selection. Further, Schevernels et al. (2014) used the electrodes P1, P2, PO3, PO4, Pz, and POz to examine how task preparation processes (for different task-difficulty levels) were related to reward prediction. While PO7 and PO8 have been most commonly used in ERP studies of the IOR effect, other electrodes were selected in the current study based on the aforementioned reward-association/reward-prediction, spatial-attention/visual-selection, and task difficulty literature, which are directly related to the aim of the current study.

**FIGURE 2:**
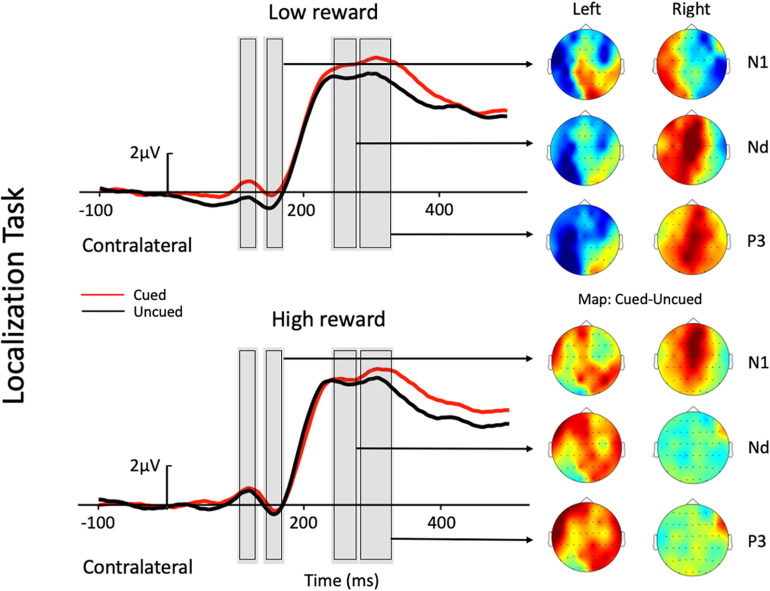
Grand average waveforms at electrode sites CP1, CP2, P1, P2, PO3, PO4, PO7, and PO8 showing the contralateral potentials produced in response to the presentation of the target in the localization task. The cued-uncued topographies of the N1, Nd, and P3 with target presented at the left and the right visual field are shown at the right panel. Positive voltage is plotted upward.

**FIGURE 3:**
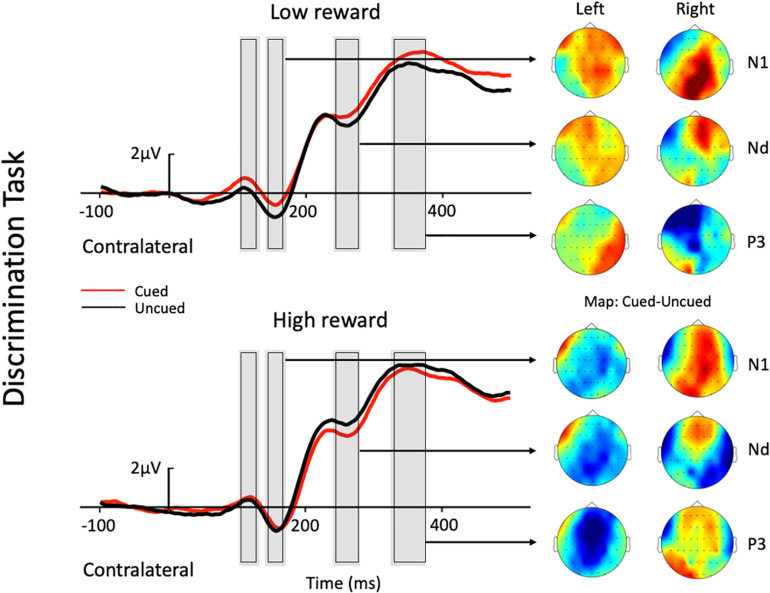
Grand average waveforms at electrode sites CP1, CP2, P1, P2, PO3, PO4, PO7, and PO8 showing the contralateral potentials produced in response to the presentation of the target in the discrimination task. The cued-uncued topographies of the N1, Nd, and P3 with target presented at the left and the right visual field are shown at the right panel. Positive voltage is plotted upward.

Mean contralateral and ipsilateral activity in the ERP were calculated for each participant for this parieto-occipital electrode pool (CP1, CP2, P1, P2, PO3, PO4, PO7, and PO8). A repeated-measures analysis of variance (ANOVA) with the factors Task (localization, color discrimination), Reward (high, low), and Cue (cued, uncued) was performed for each component: P1 (contralateral = 103–133 ms, ipsilateral = 135–165 ms), N1 (contralateral = 141–171 ms, ipsilateral = 169–199 ms), Nd (contralateral = 240–280 ms), and P3 (location task: contralateral = 281–331 ms, ipsilateral = 283–333 ms; discrimination task: contralateral = 325–375 ms, ipsilateral = 349–399 ms). For the first three components (P1, N1, and Nd), the time-windows contralateral/ipsilateral to the target location were selected according to their respective peak latencies in both the location task and the discrimination task (i.e., peak latency averaged across the two tasks), and then using a time window of 30–60 ms around the peak (depending on the shape of the curve) for statistical analyses of the mean amplitude. The peak latency for the P3 component, however, was ∼50 ms earlier in the location task than in the discrimination task (see Figures 2, 3), so using the averaged time-window of the two tasks would be inappropriate for either of the tasks. Therefore, for the P3 component, we used its corresponding time-window in each task specified above.

In addition to using the above introduced electrode pool, we also separately analyzed the earlier components (P1, N1) using just the PO7/PO8, as these are both the commonly used electrode cites for early visual components and where the P1 and N1 are usually the largest at. The averaged waveforms of PO7/PO8 across two tasks showed that the contralateral P1 peaked at 108 ms (93–123 ms) and the ipsilateral P1 peaked at 146 ms (131–161 ms), and that the contralateral N1 peaked at 160 ms (143–173 ms) and the ipsilateral N1 peaked at 194 ms (179–209 ms). Therefore, the aforementioned ANOVA analysis was also carried out on the P1 and N1 components within these specified time-windows on the PO7 and PO8 electrodes.

We set the significance level of all the ANOVAs to 0.05 and used Greenhouse–Geisser corrections for all of the effects that had two or more degrees of freedom in the numerator. We report all the repeated-measures ANOVAs with uncorrected degrees of freedom but corrected *p* values.

## Results

### Behavioral Results

#### Learning Phase

The RTs and error rates were calculated for each sub-condition according to a two (reward level: high, low) × 8 (blocks: 1, 2, 3, 4, 5, 6, 7, and 8) within-participant experimental design. Extreme responses (mean ± 3 standard deviations) were discarded from the data analyses. Less than 1% of the trials were discarded following this criterion. Mean RTs and error rates were then submitted to a 2 × 8 repeated-measures ANOVA. The results for the RTs revealed a main effect of reward level [*F*(1, 19) = 9.20, *p* < 0.01, η_p_^2^ = 0.33], with faster RTs under the high-reward condition (543 ms) than under the low-reward condition (561 ms). A main effect of block was not found [*F*(7, 133) = 1.25, *p* > 0.1], and neither was a reward level × block interaction [*F*(7, 133) = 1.32, *p* >0.1]. ANOVA of error rates did not reveal any significant results.

#### Test Phase

The RTs and error rates under each sub-condition were calculated according to the 2 (task type: localization discrimination, color discrimination) × 2 (reward level: high, low) × 2 (cue validity: cued, uncued) factorial design (see Table 1). Extreme responses (mean ± 3 standard deviations) were discarded from the data analyses. Following this criterion, 1.5% of the data were discarded. Mean RTs and error rates were then submitted to a 2 × 2 × 2 repeated-measures ANOVA. Table 1 lists the mean RTs and response error rates over participants for each experimental condition.

**TABLE 1:** RTs (ms), Error rates % (standard error), and Inversed Efficiencies (IEs) under each sub-condition.

	Reward	Discrimination task		Localization task	
	Level	Cued	Uncued	Cued	Uncued
RT	Low	518 (34)	502 (31)	362 (23)	349 (22)
	High	499 (28)	492 (28)	362 (25)	348 (23)
Error rate	Low	2.7 (0.7)	2.2 (0.6)	1.1 (0.9)	1.2 (0.9)
	High	3.6 (0.8)	2.3 (0.6)	1.1 (0.8)	1.5 (0.9)
IE	Low	530 (32)	512 (30)	369 (28)	356 (26)
	High	516 (28)	503 (27)	369 (29)	357 (28)

The results for RT (see Table 1) revealed a main effect of task type [*F*(1,19) = 92.23, *p* < 0.001, η_p_^2^ = 0.829], with the RTs in the location task (355 ms) being faster than those in the color discrimination task (503 ms). The main effect of cue validity was significant [*F*(1, 19) = 19.91, *p* < 0.001, η_p_^2^ = 0.512], with the RTs in the cued condition (435 ms) slower than those in the uncued condition (423 ms). The results also demonstrated a main effect of reward level [*F*(1, 19) = 5.12, *p* < 0.05, η_p_^2^ = 0.212], with RTs under high rewards (425 ms) being faster than those under low rewards (433 ms). No other effects reached significance.

Analysis of variance of error rates only revealed a marginally significant main effect of cue validity [*F*(1,19) = 4.122, *p* = 0.057, η_p_^2^ = 0.178], with the error rate in the cued trials (2.1%) being higher than that in the uncued trials (1.8%). The results also revealed a Task × Cue interaction [*F*(1,19) = 7.27, *p* < 0.05, η_p_^2^ = 0.277]. A simple effect analysis found no difference between the cued and uncued conditions in the location task (1.1 vs. 1.4 %), while the difference between cue validities was significant in the color discrimination task (cued vs. uncued: 3.2 vs. 2.3 %), *t*(19) = 2.54, *p* < 0.05.

Although no correlation was found between mean accuracy and RT across participants in the current study, in order to address potential concerns about a speed-accuracy trade-off in the behavioral IOR effect, inverse efficiency (IE) was calculated as the mean correct RT divided by the accuracy rate, separately for each participant and each condition (Townsend and Ashby, 1983; Kiss et al., 2009; Lee and Shomstein, 2013). ANOVA of IE revealed a main effect of task type [*F*(1,19) = 70.13, *p* < 0.001, η_p_^2^ = 0.787], with the IEs on the location task (363 ms) being faster than those on the color discrimination task (515 ms). The main effect of cue validity was significant [*F*(1, 19) = 26.33, *p* < 0.001, η_p_^2^ = 0.581], with the IEs in the cued condition (446 ms) being slower than those in the uncued condition (432 ms). The results also demonstrated a marginally significant main effect of reward [*F*(1, 19) = 3.99, *p* = 0.06, η_p_^2^ = 0.174], with the IEs under high reward (436 ms) being faster than those under low reward (442 ms). No other effects reached significance. These results, which are consistent with the RT results, again confirmed that there was no speed-accuracy trade-off to distort the observed IOR effects in the current study.

### ERP Results

**FIGURE 4:**
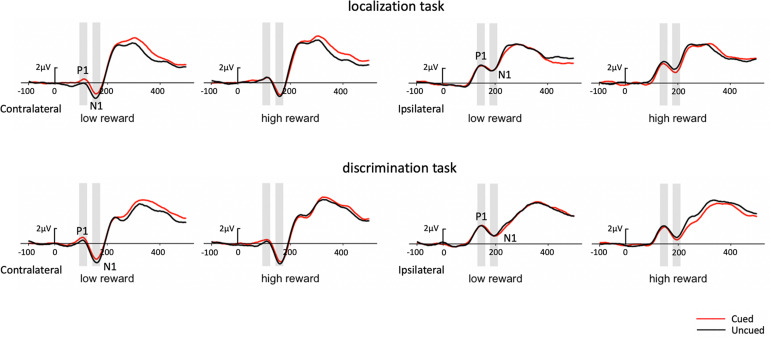
Grand average waveforms at electrode sites PO7 and PO8 showing the contralateral and ipsilateral potentials produced in response to the presentation of the target in the localization task (upper panel) and discrimination task (lower panel). Positive voltage is plotted upward.

Contralateral ERP responses time-locked to target onset from selected electrodes are depicted in Figures 2–4 for the two tasks. A brief summary of the current ERP results is listed in Table 2.

**TABLE 2:** Repeated-measures ANOVA results (based on the electrode pool of CP1, CP2, P1, P2, PO3, PO4, PO7, and PO8) summary for each ERP component contra-/ipsi-lateral to the target location.

Contralateral	Effects	P1	N1	Nd	P3
	Task	—	**	***	—
	Reward	**	—	—	—
	Cue	**	**	—	—
	Reward × Cue	Low reward: cued > uncued*** High reward: cued ∼ uncued	Low reward: cued < uncued** High reward: cued ∼ uncued	Low reward: cued < uncued* High reward: cued ∼ uncued	Low reward: cued > uncued** High reward: cued ∼ uncued

**Ipsilateral**	**Effects**	**P1**	**N1**	**Nd**	**P3**

	Task	**	**	—	**

Effects include the main effect of Task, Reward, Cue validity, and the Reward × Cue interaction. Statistical significance is marked with *0.05 < *p* < 0.1, **p < 0.05, and ***p < 0.005.

#### P1 Component

A three-factor repeated-measures ANOVA, with Task (localization, color discrimination), Reward (high, low), and Cue validity (cued, uncued), for the contralateral P1 revealed a main effect of reward type, [*F*(1,19) = 4.39, *p* = 0.050, η_p_^2^ = 0.188], with amplitudes elicited by high reward target higher than those elicited by low reward target (0.63 vs. 0.34 μV), and a main effect of cue validity [*F*(1,19) = 6.29, *p* = 0.021, η_p_^2^ = 0.249], with the amplitudes elicited under cued trials higher than those under uncued trials (0.69 vs. 0.28 μV). The results also showed a significant Task × Reward interaction [*F*(1,19) = 5.15, *p* = 0.035, η_p_^2^ = 0.213], and a significant Reward × Cue interaction [*F*(1,19) = 9.17, *p* = 0.007, η_p_^2^ = 0.325]. Simple effect analysis, to follow up on the Task × Reward interaction, showed that the amplitude of the P1 elicited in the high-reward condition was greater than that in the low-reward condition (0.80 vs. 0.16 μV) in the localization task [*t*(19) = 3.09, *p* = 0.004], while the amplitude of the P1 in the high- and low-reward conditions exhibited no difference in the discrimination task (0.46 vs. 0.51 μV) [*t*(19) < 1]. The simple effect analysis, to follow up on the Reward × Cue interaction, showed that in the low-reward condition, the amplitude of the P1 elicited in the cued condition was more positive than that in the uncued condition (0.69 vs. −0.02 μV) [*t*(19) = 3.72, *p* < 0.001]; in the high-reward condition, however, the amplitude of the P1 in the cued and uncued (0.68 vs. 0.58 μV) conditions exhibited no difference [*t*(19) < 1].

The same ANOVA on the ipsilateral P1 revealed a significant main effect of Task [*F*(1,19) = 4.67, *p* = 0.044, η_p_^2^ = 0.197], with the amplitudes elicited in location task higher than those in the discrimination task (2.72 vs. 2.35 μV).

In addition, as early components have typically been reported at PO7/PO8, we performed the same analysis on P1 with just the PO7/PO8 electrodes (see Figure 4 for waveforms at PO7/PO8). The three-factor repeated-measures ANOVA of the contralateral P1 component revealed a marginal cueing effect [*F*(1,19) = 4.06, *p* = 0.058, η_p_^2^ = 0.176], with the amplitudes elicited under cued trials higher than those under uncued trials (0.65 vs. 0.37 μV). Results also revealed a significant Task × Reward interaction [*F*(1,19) = 8.13, p = 0.010, η_p_^2^ = 0.300]. Simple effects analysis showed that the amplitude for high-reward was greater than that for low-reward (0.68 vs. 0.23 μV) in the localization task [*t*(19) = 2.49, *p* = 0.017], while the amplitude did not differ between reward levels in the discrimination task (0.44 vs. 0.68 μV) [*t*(19) = −1.38, *p* = 0.176]. Moreover, a significant Reward × Cue interaction [*F*(1,19) = 4.91, *p* = 0.039, η_p_^2^ = 0.205] demonstrated that the P1 amplitude was higher in the cued than in the uncued trials (0.69 vs. 0.23 μV) under low reward condition [*t*(19) = 2.87, *p* = 0.007], while no difference was found between the cued and uncued conditions under high reward condition (0.60 vs. 0.51 μV) [*t*(19) < 1].

The same ANOVA on the ipsilateral P1 revealed no significant effects.

#### N1 Component

The three-factor repeated-measures ANOVA of the contralateral N1 component revealed a main effect of task type [*F*(1,19) = 7.76, *p* = 0.012, η_p_^2^ = 0.290], with the amplitude of the N1 elicited in the color discrimination task being more negative than that in the location task (−1.12 vs. −0.50 μV). A main effect of cue validity was also found [*F*(1,19) = 6.2, *p* = 0.023, η_p_^2^ = 0.245], with the amplitude elicited for uncued locations being more negative than those for the cued locations (−1.02 vs. −0.59 μV). Although the Reward × Cue interaction [*F*(1,19) = 1.87, *p* = 0.187, η_p_^2^ = 0.090] did not reach significance, a planned simple effect analysis discovered that the amplitude of the N1 elicited in the uncued trials was more negative than that in the cued trials (−1.11 vs. −0.49 μV) in the low-reward condition [*t*(19) = 2.79, *p* = 0.008], while the amplitude of the N1 in the uncued and cued trials exhibited no difference under the high-reward condition (−0.94 vs. −0.70 μV) [*t*(19) < 1].

The same ANOVA on ipsilateral N1 revealed a main effect of task type [*F*(1,19) = 6.88, *p* = 0.017, η_p_^2^ = 0.266], with the amplitude of the N1 elicited in the color discrimination task being more negative than that in the location task (1.53 vs. 2.32 μV).

In addition, we performed the same analysis on N1 with the PO7/PO8 electrodes (see Figure 4 for waveforms at PO7/PO8). The three-factor repeated-measures ANOVA of the contralateral N1 component revealed a main effect of task type [*F*(1,19) = 7.19, *p* = 0.015, η_p_^2^ = 0.274], with the N1 amplitude elicited in the color discrimination task being more negative than it was in the localization task (−2.61 vs. −1.89 μV). A main effect of cue validity was also found [*F*(1,19) = 5.88, *p* = 0.025, η_p_^2^ = 0.236], with the amplitude elicited for the uncued targets being more negative than those for the cued targets (−2.47 vs. −2.03 μV). Although the Reward × Cue interaction did not reach significance, we examined the cueing effects separately for low and high reward conditions based on our planned comparisons. The results revealed that uncued trials elicited greater N1 than cued trials (−2.49 vs. −1.98 μV) under low reward condition [*t*(19) = 2.32, *p* = 0.027], while the N1 amplitude in the uncued and cued trials (−2.44 vs. −2.08 μV) exhibited no difference under high reward condition [*t*(19) = 1.65, *p* = 0.108].

The same ANOVA on the ipsilateral N1 revealed no significant effects.

### Nd Component

The three-factor repeated-measures ANOVA on the Nd component revealed a main effect of task type [*F*(1,19) = 98.9, *p* < 0.001, η_p_^2^ = 0.611], with the amplitude of the Nd elicited in the color discrimination task being more negative than that in the location task (3.90 vs. 6.32 μV). The results also revealed a Reward × Cue interaction [*F*(1,19) = 4.21, *p* = 0.054, η_p_^2^ = 0.181]. Simple effect analysis to follow-up on the Reward × Cue interaction discovered that the amplitude of the Nd elicited for the uncued condition was more negative than that for the cued condition (4.76 vs. 5.27 μV) when the reward level was low [*t*(19) = 1.84, *p* = 0.074], while the amplitude of the Nd in the cued and uncued conditions exhibited no difference under the high-reward condition (5.12 vs. 5.29 μV) [*t*(19) < 1].

#### P3 Component

Three-factor repeated-measures ANOVA on the P3 component revealed a significant Reward × Cue interaction [*F*(1,19) = 5.37, *p* = 0.032, η_p_^2^ = 0.220]. Simple effect analysis discovered that in the low-reward condition, the amplitude of the P3 elicited for the cued condition was greater than that for the uncued condition (6.96 vs. 6.32 μV) [*t*(19) = 2.41, *p* = 0.023]; in the high-reward condition, however, the amplitude of the P3 for the cued and uncued (6.93 vs. 6.83 μV) conditions exhibited no difference [*t*(19) < 1].

The same ANOVA on the ipsilateral P3 revealed a main effect of task type [*F*(1,19) = 5.35, *p* = 0.032, η_p_^2^ = 0.220], with the amplitude of the P3 elicited in the color discrimination task being greater than that in the localization task (7.01 vs. 6.07 μV). No other effects approached statistical significance.

## Discussion

In order to explore whether target-reward association can modulate the rather automatic mechanism of IOR, the current study used a spatial cueing paradigm, with targets shown in colors previously associated with either high or low rewards. Though the behavioral data only exhibited an IOR effect, ERP recordings demonstrated an interaction between reward level and cue validity, with differences between the cued and uncued trials (i.e., neural patterns of IOR) for the P1, N1, Nd, and P3 under low reward condition, which were diminished under high reward condition. Participants were given a color discrimination task and a localization task to examine whether the interaction between reward and IOR was affected by task relevance. While the results showed that this interaction was not affected by task relevance, the type of task appeared to separately interact with reward level for the P1 component, where the difference in P1 amplitude between high and low reward was observed only in the location task (not in the color discrimination task). In addition, the present study examined the four most important ERP components in the exogenous cueing paradigm (i.e., P1, N1, Nd, and P3) in an effort to contribute to search for a stable ERP marker of IOR.

In the learning phase, RTs for high-reward associated targets were significantly faster than those for low-reward associated targets, which means participants learned the association between available rewards and particular colors. This is consistent with earlier studies that demonstrated an association between a stimulus and rewarding information can be established by training (Raymond and O’Brien, 2009; Anderson et al.,2011a,b, 2012, 2013; Anderson, 2013). In the test phase, RTs for the cued condition were slower than those for the uncued condition, exhibiting IOR in both high- and low-reward conditions for both the color discrimination task and localization task. RTs in the high-reward condition were faster than those in the low-reward condition, indicating an attentional bias toward high-reward associated information, which was more profound in the color discrimination task than in the localization task, though both reached significance. Although the interaction between reward and cueing was not significant in the behavioral data, the ERP components revealed differential patterns between cued and uncued trials for the low-reward condition and high-reward condition, revealing underlying early neural processing mechanisms before the final behavioral outcome.

As described in the Introduction, previous studies have often reported an IOR effect, with a reduced P1 component for cued vs. uncued trials (to index impaired early perceptual processing for cued location at long SOAs), although P1 modulation had been observed without a behavioral IOR effect, and vice versa. In the current study, we found an anomalous cueing effect pattern on the P1 component, with an enhanced P1 for cued trials vs. uncued trials. This was quite unexpected if P1 is an index of early perceptual processing at a long SOA (typically 500–1,000 ms, which the current study falls within), although this pattern of P1 cueing effect was also reported in another study (Lubbe et al., 2005). Lubbe et al. (2005) reported an unusual behavioral faciliatory effect at a long SOA (940 ms) that was associated with significant P1 enhancement for cued vs. uncued trials in a discrimination task. While such an anomalous pattern in their study could have resulted from the interference effect from their experimental conditions (i.e., a set of different SOAs) or from their task demand, in the current study, the associations between the target and colors, which were previously associated with either high or low reward, could have contributed to our observed pattern of the P1 cueing effect. Alternatively, the P1 difference might be explained, rather, by the following N1 for uncued trials. More specifically, the peak of P1 and the onset of N1 were close in time, and the amplitude difference between the cued and uncued trials on N1 seemed to have started from P1, lasting all the way to N1 (as shown in the figure). Therefore, it might be that relatively smaller P1 amplitude for uncued trials is caused by an already initiated stronger negativity for the uncued trials vs. the cued trials. Due to the anomalous pattern of P1 modulation, we will focus our discussion mainly on the results for the N1, Nd, and P3 components.

A modulated N1 has been suggested to represent IOR by a string of studies, with larger N1 amplitudes for uncued trials than cued trials (e.g., Prime and Ward, 2004, 2006; Tian and Yao, 2008; Prime and Jolicoeur, 2009; Gutiérrez-Domínguez et al., 2014; Satel et al., 2014;, but see e.g., McDonald et al., 1999; Satel et al., 2012; Martín-Arévalo et al., 2014). In the present study, the absent cueing effect on the N1 contralateral to the target location in high-reward trials was observed under low reward. Specifically, under the low-reward condition, the target-elicited N1 amplitude was reduced under the cued trials compared to the uncued trials; however, when the target was previously associated with high reward, no difference was found between the cued and uncued conditions. The absence of cueing effects in high-reward trials suggests that the target-reward association may be resistant to IOR on the N1 component, depending on the reward level. Previous studies reported that an enhanced N1 was usually found for attended stimuli, and that the component reflected early perceptual discrimination processing of the attended stimuli (Vogel and Luck, 2000; Boksem et al., 2005). This may suggest that the low-reward associated target did not impede the inhibitory effect on stimulus discrimination processes at the cued location. However, high-reward associated targets enjoyed prioritized processing, and thus, had modulated the attentional inhibition at the cued location.

The Nd component in the time-period between 240 and 280 ms post-stimulus is recognized as a negative difference between cued and uncued trials (e.g., Eimer, 1994). A reduced Nd amplitude for cued than uncued trials has been traditionally associated with IOR (e.g., McDonald et al., 1999; Hopfinger and Mangun, 2001; Prime and Ward, 2004, 2006; Prime and Jolicoeur, 2009; Satel et al., 2012, 2014; Gutiérrez-Domínguez et al., 2014; Martín-Arévalo et al., 2016). In the present study, a modulated Nd contralateral to the target location was also found under the low-reward condition, with targets under the uncued condition eliciting a larger Nd amplitude than that under the cued condition (i.e., IOR). Whereas, under the high-reward condition, Nd amplitudes exhibited no difference between cued and uncued trials. Therefore, the current results revealed that the Nd component contralateral to the target location also exhibited resistance to IOR under high reward. Previous studies indicated that the Nd reflects the processing of the attended stimuli (Eimer, 1993, 1994). Thus, when IOR appeared in the low-reward condition due to the inhibitory effect at the cued location, the Nd amplitude under the cued condition was significantly smaller than that under the uncued condition. However, when the target was associated with high reward, attentional bias toward the high-reward associated information weakened the inhibitory effect at the cued location, leading to an undifferentiated amplitude between the cued and uncued conditions.

The P3 enhancement for cued compared to uncued trials was associated with IOR in some previous studies (McDonald et al., 1999, Experiment 1; Prime and Jolicoeur, 2009), although most previous studies have not tested modulations of this component. In the present study, a modulated P3 contralateral to the target location was observed under the low-reward condition, with greater amplitudes for cued trials compared to uncued trials. Since P3 is particularly sensitive to target expectancies, and its amplitude is usually larger for novel stimuli (e.g., Donchin, 1981; Friedman et al., 2001), McDonald et al. (1999) proposed in his study that one interpretation of the observed P3 pattern was that cued targets were unexpected, even though they occurred with the same frequency as uncued targets. This is to say that IOR inhibits attention at the previously cued location, and thus, one is more likely to expect targets to appear at the uncued location. This helps to explain why the present study found a greater P3 for cued compared to uncued trials under low reward. In this sense, the P3 may also serve as a valid ERP marker for IOR, though further studies should be carried out to test this hypothesis about the P3.

On the other hand, this may also help to explain the modulated pattern under high reward in the present study, where P3 amplitudes exhibited no difference between the cued and uncued conditions. A possible interpretation is that high-reward associated targets automatically attracted more attention, which reduced the unexpectedness/novelty at the cued target location. In other words, high-reward information drew participants’ attention to the ought-to-be unexpected location so that when participants were supposed to focus fully on the uncued location due to inhibition at the other, they were now inevitably drawn to the cued location at the same time. Thus, expectedness became generally equal at both locations, which is to say that the cued location was no longer novel to the participant. Therefore, the combined effect of reward prioritized processing and inhibition at the cued location, or reorientation toward the uncued location, resulted in no observable difference between P3 amplitudes in the cued and uncued trials under the high-reward condition. This reflects that the inhibition at the cued location, i.e., IOR, was effectively modulated by reward association.

The present experiment did not observe a significant three-way interaction between task type, reward level, and cue validity on any of the ERP components. However, an interaction between task type and reward was found for the P1 component. Briefly, we observed a task by reward interaction in which high reward elicited a larger P1 component than low reward in the localization task, but this reward effect was not observed in the color discrimination task. One possible explanation is the complexity of task demands. When a task is easier, as in the location task, more early perceptual resources can be spared to allocate to other factors, and in our case the reward level, whereas the harder the task demands are, the less perceptual-attentional resources can be spared for task-irrelevant factors. Given the anomalous pattern of the P1 cueing effect, we are hesitant to make further interpretations regarding the results found for the P1 component.

Overall, the result patterns for the N1, Nd, and P3 components in the present study provide electrophysiological evidence supporting that a target-reward association in an exogenous cueing paradigm can effectively modulate the neural process of IOR in such a way that a low-reward associated target is subject to IOR while a high-reward associated target is resistant to IOR. Over the decades, a wealth of studies was conducted to investigate the nature of IOR, with many results indicating that IOR is mainly stimulus-driven and automatic. Taylor and Therrien (2005) used intact-face vs. identical scrambled-face cues to elicit IOR, with the expectation that with emotional valence controlled at neutral, attention would be differentially attracted to and/or maintained on intact-face cues vs. scrambled-face cues. However, the results disclosed that IOR was not affected by either cue or target configuration. Therefore, Taylor and Therrien concluded that IOR is a “blind” mechanism that is exempt from the influence of biologically relevant cues and target stimuli. Wang et al. (2010) supported this notion with results of their study. Using upright vs. reversed-face cues, Wang et al. (2010) found that IOR was not affected by face orientation. As a matter of fact, Theeuwes (1994, 2010) has long argued that the capture of attention and the following inhibition after an abrupt appearance of an object is independent of the participant’s personal beliefs and goals. However, the results of the current study suggest that IOR is not a “blind” mechanism, and that the automatic process of IOR can be effectively modulated by the prioritized processing of reward through target-reward association.

In addition, unlike earlier studies examining reward’s effect on IOR, where IOR was not observed in the control group, the neural pattern of IOR observed for all three of the components (N1, Nd, and P3) under low reward condition in this study provide an optimal control condition for the investigation of high reward’s effect on IOR. Therefore, the diminished cue—uncued difference under the high-reward condition demonstrates that the more automatic process of attention prioritizing *via* reward-target association can be resistant to IOR, impeding inhibition at the cued location by guiding attention to the high-reward associated target location, and that such a modulatory effect is not affected by task relevance, as previously discussed. Moreover, the results under low rewards suggest that the P3, a component that has rarely been associated with IOR effects in previous studies, may serve as a potential valid IOR marker, though future studies are needed to examine the process the P3 represents that underlie IOR effects.

## Conclusion

The present study demonstrated that IOR is not a “blind” mechanism. Electrophysiological evidence of a reward-by-cueing interaction shows that target-reward associations can effectively modulate the rather automatic mechanism of IOR in such a way that even though low-reward associated targets are subject to IOR, high-reward associated targets are resistant to IOR. This is most likely because the automatic prioritized processing of high-reward associated targets impede attentional inhibition at the cued location (i.e., target location). The underlying neural mechanism was explored using ERP, with the N1, Nd, and P3 components all demonstrating a modulatory effect of reward on IOR.

In addition, the present study provides further evidence relevant to the search for an electrophysiological marker for IOR. Enhanced N1 and Nd amplitudes for uncued compared to cued trials, and an enhanced P3 amplitude for cued vs. uncued trials were patterns observed under low reward in the present experiment, which had been suggest by prior studies related to IOR effects. While the P3 cueing effect seems promising from the current results, future studies should be carried out to investigate the effectiveness of P3 in association with IOR, particularly, the pattern of P3 enhancement/reduction for cued vs. uncued.

## Data Availability Statement

The raw data supporting the conclusions of this article will be made available by the authors, without undue reservation.

## Ethics Statement

The studies involving human participants were reviewed and approved by The Ethics Committee of the School of Psychology at Capital Normal University. The patients/participants provided their written informed consent to participate in this study.

## Author Contributions

All authors listed have made a substantial, direct and intellectual contribution to the work, and approved it for publication.

## Conflict of Interest

The authors declare that the research was conducted in the absence of any commercial or financial relationships that could be construed as a potential conflict of interest.

## Footnotes

1 https://www.neurobs.com/
